# An evolution of low-field strength MRI

**DOI:** 10.1007/s10334-023-01104-z

**Published:** 2023-06-08

**Authors:** Juergen Hennig

**Affiliations:** Department of Radiology, Medical Physics, Faculty of Medicine, University of Freiburg, Killianstr.5a, 79106 Freiburg, Germany

**Keywords:** MRI, Low-field MRI, Ultralow-field MRI, MRI history, MRI technology

## Abstract

The paper describes the evolution of low-field MRI from the very early pioneering days in the late 70 s until today. It is not meant to give a comprehensive historical account of the development of MRI, but rather to highlight the different research environments then and now. In the early 90 s, when low-field systems below 1.5 T essentially vanished, there were just no reasonable means available to make up for the factor of roughly three in signal-to-noise-ratio (SNR) between 0.5 and 1.5 T. This has drastically changed. Improvements in hardware—closed Helium-free magnets, RF receiver systems and especially much faster gradients, much more flexible sampling schemes including parallel imaging and compressed sensing and especially the use of AI at all stages of the imaging process have made low-field MRI a clinically viable supplement to conventional MRI. Ultralow-field MRI with magnets around 0.05 T are also back and constitute a bold and courageous endeavor to bring MRI to communities, which have neither the means nor the infrastructure to sustain a current standard of care MRI.

## Introduction

The following overview is meant to be more illustrative than comprehensive. The main purpose is not so much to give a full historical overview—which would require a book rather than a journal article—but to illustrate, how and why low-field MRI vanished already in the 80’s and what has changed since then to make this an attractive prospect after all this time.

MRI began at very low-fields, the initial home-built pioneering magnets operated at field strength around 0.05 T. First commercial systems, which appeared in the early 80’s went up to ~ 0.5 T. The development was more or less abruptly terminated by the introduction of the first 1.5 T scanner by GE in 1983, which set the bar for high-field MRI and has been the mainstay of clinical MRI since then. Low-field MRI continued to exist in the form of permanent magnet systems, which were, however, considered as low-cost, low-performance systems for those who could not afford high field systems. Some groups continued to work on ultra-low-field MRI with the goal to make MRI accessible also outside those regions which can afford the still costly standard MRI system. Only quite recently recognition dawned in the wider research community that MRI is still a very costly modality and—in a global context—only accessible to the privileged few living in high income parts of high income countries. This initiated a rapid development at both ‚traditional' low field strength regimes: ultra low-field MRI (ULF-MRI) at ~ 0.05 T with many groups working on different designs and design goals, and low-field MRI (LF-MRI) at around 0.5 T.

In the following I will briefly go through the very early pioneering days followed by a look at the more recent developments of ULF- and LF-MRI.

### Pioneering days

I have started working in MRI in 1984, so not having been a direct witness of the early days I don’t claim any authority for a definite account, but I clearly recall, that the idea that NMR could be used for medical diagnosis caused a lot of excitement way beyond the NMR community.

Reading through the testimonies of the early pioneers it becomes clear that these were very intense and exciting times and it would be a gargantuan task to try to unravel the quite complex developments at the various sites working on making MRI happen.

There are numerous accounts about the early days of MRI [1, 2]. While personal accounts, which have been published and/or recorded by the ISMRM Historical Archives Committee shed some light onto the personalities doing the work, I still very much recommend reading the original papers although it needs to be noted that many developments had been presented at various meetings of the still small MRI community but never been properly published.

Paul Lauterbur’s seminal paper [3] immediately created a lot of excitement in and outside of the NMR-community. The possibility of making images with the ability to show soft tissues rather than just bones as in conventional x-ray radiography inspired several scientists to investigate, how this exciting discovery can be realized in practice.

**Fig. 1:**
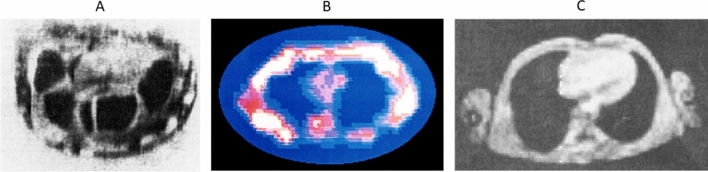
Early MR images: **A** first MRI image of a human wrist (from Fig. 1, ref. [8]) acquired with the sensitive line method on a 0.7 T magnet in 9 min. **B** Image of a human thorax acquired with the FONAR method (from ref. [9]). The image was acquired in a superconductive magnet with 0.0508 T by moving the subject to 64 different positions inside of a huge magnet, total acquisition time was 4 h 45 min. **C** Spin-warp image from a human thorax (from Fig. 2 ref. [17]) acquired at 0.04 T in 64 s

In order to appreciate the developments at this time, one has to realize the scientific environment, which was very different from today. The widespread use of computers had only just begun, some so-called minicomputers had already been introduced, where minicomputer is a somewhat misleading term. As an example the PDP-11 as one of the most successful systems had a weight of 34 kg and a size of roughly 60 × 70 × 25 cm. Such instruments were not yet in common use, most academic institutions had to rely on centralized mainframe computers. Image reconstruction of Lauterbur’s Zeugmatography-method—which today is more commonly called radial encoding—was performed on a mainframe computer using ART(algebraic reconstruction technique) [4]. Acquired data were transferred via punch card or tape to a mainframe computer, reconstructed pixel values were visualized using some homemade routines [5]. Although radial encoding is an efficient imaging scheme and still very much in use today, it was then hardly used due to the cumbersome reconstruction process. When Hinshaw published his sensitive point method [6], he stated: ‘…*This (Lauterbur’s) method of producing an image requires large signal handling and computing facilities, particularly if useful resolution is to be obtained*…’ as one of the motivations for his method. In 1977 his group was able to produce the very first human in vivo-image of a wrist (Fig. 1A) using a homebuilt 30 MHz system based on a 0.7 T Varian electromagnet with a 10 cm bore [7, 8]. The sensitive line method he used was not very efficient and data acquisition took about 9 min for a single image. In the same year Damadian published his first image of a human thorax (Fig. 1B) acquired using a superconductive (‘supercon’) magnet with a field strength of 0.05 T corresponding to 2.3 MHz resonance frequency [9]. Spatial localization is reported to be achieved using the Field Focusing Nuclear Magnetic Resonance (FONAR) method. The method had been published previously [10, 11], but the experimental description is not overly clear (…*This is accomplished by shaping the magnetic fields (H0 and H1) across the entire sample so as to construct a small resonant window within the sample, such that the ratio of the spin moment of the nucleus to its gyric moment is everywhere satisfied by the static and time varying H fields and is everywhere dissatisfied beyond its boundaries…*). The description of spatial localization in the FONAR-patent [12], which is also cited in the paper, is even less clear. The concept seems to be reminiscent of inverse radar (‘…*A transmitter probe is provided with a beam focusing mechanism for focusing the radiated magnetic energy from the radio frequency generator into a beam having a narrow cross-section*….), but it remains unclear how such beam focusing should work at a wavelength of several meters. In the end the image seems to have been reconstructed from measuring the signal from 106 different locations within the body acquired by moving the subject around in the magnet with a total measurement time of 4 h 45 min [13].

Lauterbur also tried to translate his ideas into practice, but with limited success. For lack of funding the ‘Big Red’ magnet he acquired had an open diameter of only 42 cm once RF- and gradient coils were in, which was too small for humans [5]. There don’t seem to be any publications where this magnet was used, which indicates, that the small size was not the only problem of his setup.

The most influential development was undoubtedly the effort of the Aberdeen group led by John Mallard [14–16]. His magnet had a field strength of 0.04 T, which was created by a vertical electromagnet with 4 magnet coils in a Helmholtz configuration. Key to the success was on one hand the use of the spin warp technique invented by Bill Edelstein [17] from his group (Fig. 1C). The spin warp technique used the principles of the NMR Zeugmatography published in 1974 by Kumar and Ernst [18], but it replaced the variable time used for stepping through the phase encoding step by a variable gradient applied at a constant time. In retrospect this seems to be a trivial modification, but at that time it was a given, that two-dimensional FT-NMR techniques had to have two temporal dimensions, one direct, one indirect. To replace the indirect temporal dimension by a variable gradient thus was a conceptional quantum leap. Using a constant echo time removed the sensitivity of the original method to T2*-decay, the resulting images looked—finally—like ‘real’ MR-images.

The three methods described used a single-line (Hinshaw), single point (Damadian) and 2D-approach (Edelstein). Already in 1979 P. Bottomley showed that 2D-techniques are most efficient compared to the other two [19], which is reflected by the difference in acquisition time. 3D would be even more efficient, but wasn’t yet known at that time.

The second and key factor to success was to make the system available to patients, which were brought over from Aberdeen Royal infirmary in order to do some real clinical evaluation. This turned out a huge success and paved the way towards commercialization of MRI.

Reading through the narrations of the early pioneers one cannot help but note that on top of the scientific competition there were also quite a few clashes between the personalities involved, which just shows, that yes, science is pure and beautiful, but scientists are just like other human beings. The elephant in the room is the feud between Lauterbur and Damadian, which seems to have started very early on [13]. It is noted that Lauterbur mentions in his original paper that MRI may be useful for cancer detection, but for this he cites a paper from 1972 by Weisman [20] and omits a reference to the earlier Science publication by Damadian [21]. Likewise, Damadian in his publication of the image of a human thorax gives reference to the ‘Fourier NMR Zeugmatography’ from Kumar and Ernst [18] but omits to reference Lauterbur’s earlier publication.

It is most remarkable that the two imaging techniques which led to a profound change in clinical diagnostics—CT and MRI—appeared only a few years apart from each other. Both used the same principle for making an image—projection reconstruction—but were invented to all our knowledge totally independently and had a totally different pathway into clinical practice. For MRI it took a lot of development in methods and technologies to put the principles into reality and took nearly a decade from the initial publication to the first commercial scanners in 1980. For CT the first commercial equipment actually preceded the first proper scientific publication. Godfrey Hounsfield had the idea already in 1967. He was employed by EMI and given the green light to develop an actual scanner. EMI was basically a record company and tried to branch out into other areas of business. It has been said, that the development of CT took place thanks to the royalties EMI received from selling millions of Beatles records. This is not exactly true, the CT project was mostly funded through a grant from British Department of Health and Social Security (DHSS) [22]. On the other hand, without the comfortable financial position of EMI thanks to the Beatles, EMI would have hardly employed a free-wheeling spirit like Godfrey Hounsfield to realize his dream. Interestingly, Sir Godfrey joined the MRI group at Royal Brompton during his retirement and co-authored some papers with David Firmin [1, 23–25].

### MRI becomes clinical

In spite of the existing reservations in the scientific community, that MRI was more a laboratory curiosity than a clinically viable concept, industry started very early to look into this new technology. Ian Young was tasked by EMI to develop a NMR imaging machine, although he—according to his own testimony [1]—had at that time no prior knowledge about NMR. He still built up a team, which successfully developed a system with a 0.3 T superconductive magnet (the 2nd supercon after Damadian’s), which was eventually modified and installed at Hammersmith Hospital working at 0.15 T.

A bit later Larry Crooks and Leon Kaufman worked on a supercon magnet originally bought by Pfizer, which later was sold to Diasonics [1, 2]. They established a strong collaboration with Alex Margulis at UCSF. Already in 1982 they published a review paper about the status of MRI showing good images from all over the body acquired by various groups [26].

The first commercial scanners already appeared in 1980 and the market quickly proliferated. Systems were distinguished by the magnet technology used—resistive, permanent, and superconductive—with a typical field strength below 0.5 T.

The advent of clinical MRI was facilitated by the rapid development of computers. It was not only the progress in computing power offered by minicomputers like the already mentioned PDP-11 or VAX-11. The DEC UNIBUS^™^ used by PDP (and others) also offered a standardized interface to drive the various other hardware components necessary to perform MRI.

Already in the very early days most scanners used the spin-warp technique [17] for Cartesian sampling for its very benign artifact behavior compared to the more sensitive radial encoding. Cartesian sampling is more forgiving for gradient imperfections, whereas radial encoding requires exact control especially with respect to gradient delays. In Cartesian sampling motion artifacts which invariably occur in body imaging at the long acquisition times at that time lead to doubling of contours along the phase encoding direction. Radiologists quickly learned to ‘see through’ this artifact, whereas the streaking artifacts caused by imperfections in radial sampling may lead to artifacts, which may occur anywhere in the image and may easily lead to misdiagnosis. Furthermore and most importantly from a practical point-of-view Cartesian sampling allows to reconstruct images with the Fast Fourier Transform algorithm [27] on fast array processors. In a product brochure from Diasonics from 1984 reconstruction time is given as 3 s for a 128 × 128 image and 6.5 s for 256 × 256.

**Fig. 2:**
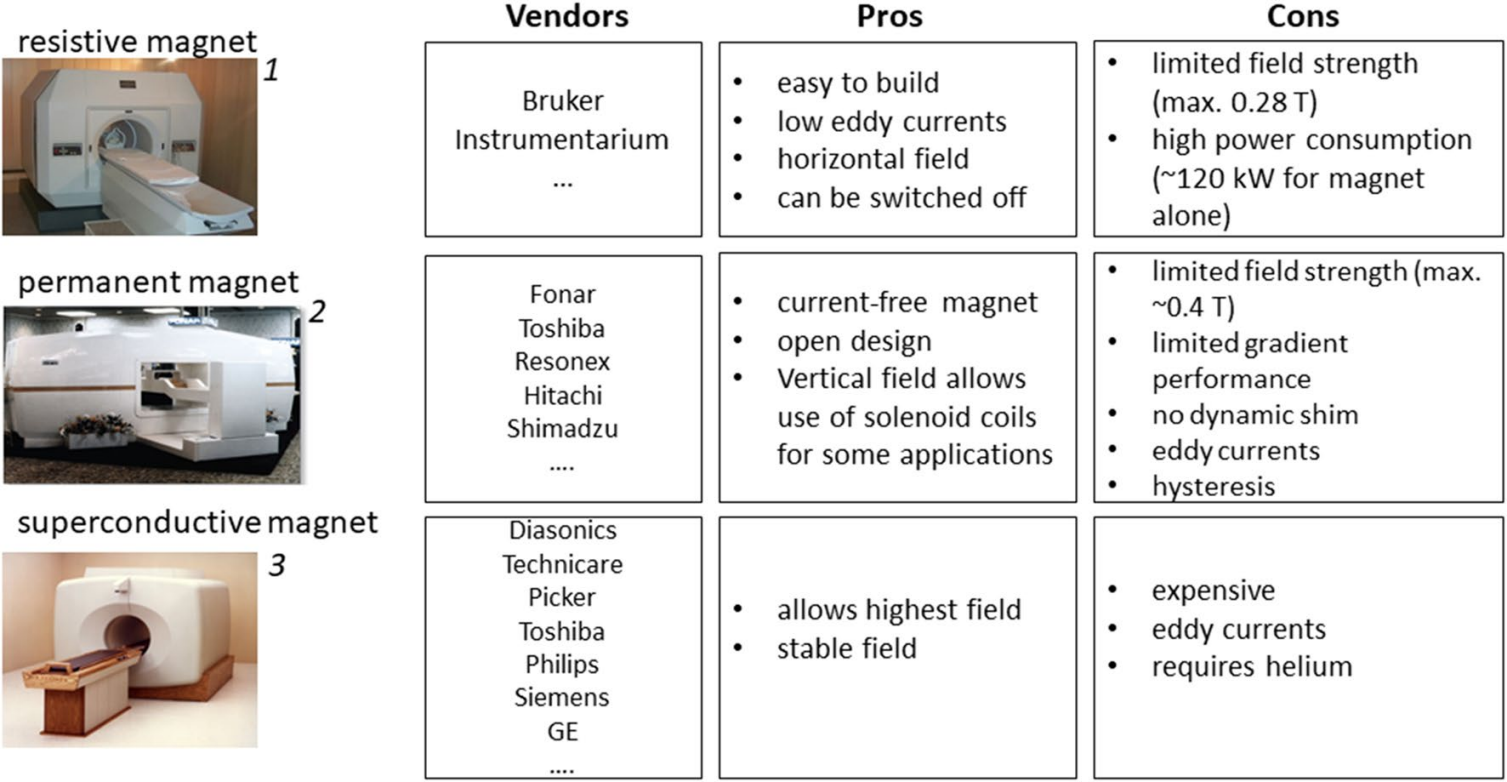
Schematic overview of clinical MRI in the early 80 s, showing some of the main vendors of MRI with resistive, permanent and superconductive magnets, and the respective pros and cons of the different technologies. Pictures: **1**: Bruker R28, courtesy Bruker Biospin, Germany, **2**: Fonar QED80 from https://fonar.com/news/historical-photos.html, **3**: UC San Francisco, Library, Special Collections. https://calisphere.org/item/3ec35cdd-85b8-4433-af08-e97dfee781cf/

Figure 2 gives a schematic overview of the market in the early 80’s with some keywords on the pros and cons of the different magnet technologies.

Resistive magnets were the most easy to build and were abundantly used by the early pioneers. Resistive magnets also have the advantage of a very beneficial eddy current behavior. They do not have extended metal surfaces to start with and at room temperature any eddy currents will fade away very quickly. Magnets can be switched on and off rather rapidly to reduce power consumption. Disadvantages of resistive magnets lie in the limited field strength. The highest field in a commercial whole body magnet was 0.27 T (Tomikon R27, Bruker, Germany). Electric power consumption at such fields was ~ 120 kW for the magnet alone, which clearly precludes such systems in our age of awareness for the carbon footprint—driving such magnet with sustainable energy would require more than 1000 m^2^ of solar cells.

Permanent magnets have been at first sight a very attractive option. Once the magnet is built, the magnet field comes for free. Permanent magnets also allow an open and patient friendly design. The vertical field allows the use of solenoid coils, which helps to improve SNR compared to the resonators used for magnets with horizontal field orientation. Field strength is also somewhat limited to ~ 0.4 T for whole body systems. The main drawback regarding the stability of such a magnet is the strong temperature dependence of the magnetic field. Around room temperature the temperature coefficient of magnetite is ~ 1000 ppm per degree K, which necessitates extremely exact temperature stabilization for stable operation, which is somewhat at odds with the use of such systems in underdeveloped areas. Even worse for imaging purposes are eddy current problems and especially hysteresis effects, which make such systems very problematic for use with phase sensitive sequences like RARE(TSE, FSE,..). The open geometry with horizontal poles is nice with respect to patient access, but makes it challenging to integrate shims or shielded gradients.

Given the problem of these conventional magnet designs it is no surprise that supercons have been dominant in the market from early on. Although the cryostat was originally also problematic with respect to its eddy current behavior, this problem has been solved in the early 90 s with the advent of shielded gradients.

The low-field typically used in the early 80 s was not only due to technological limitations. In 1978 Paul Bottomley had published a theoretical analysis of the interaction of RF fields with a human body [28] and demonstrated, that beyond 30 MHz phase distortions will occur and penetration depth will suffer. In 1979 Hoult and Lauterbur published a paper with an analysis of the noise behavior in humans [29] and concluded “*… that the frequency of operation of the spectrometer with human samples should be less than about 10 MHz*”. An excerpt from a Diasonics-brochure from 1984 demonstrates that these papers had been taken seriously:

*….At the same time we discovered many factors which demonstrated that operating above 5.0 kGauss would create substantial problems in resolving power, in the environment and in handling patients. Therefore we selected a magnet designed for 5.0 kGauss and an operating magnetic field of 3.5 kGauss*…. (from: A New Technology Conquers the World. Diasonics Inc.).

In view of this scenario the introduction of a 1.5 T system by GE in 1983 marked a disruptive milestone and led to a total reassessment of the further development goals. Other vendors followed quickly with their own 1.5 T systems. It should be noted that there was nothing wrong with the science of the earlier publications, it was just that the conclusions drawn from the results were overly cautious.

Low-field systems did not vanish immediately. The ‘field-strength war’ [2, 30] raged until the early 90 s. Most of the main vendors offered systems at lower fields (0.5, 0.8, 1 T) in addition to their 1.5 T ‘high-end’ systems. There were quite a number of publications all coming to the same conclusion that, yes, high field has higher SNR compared to low-field, but there is no statistically significant improvement" in diagnostic efficiency [31–37]. Still—as my then radiologist colleague H. Friedburg used to paraphrase Voltaire—‘the better image is the enemy of the good image’ and 1.5 T won out in the end.

The trend towards high fields was also a contributing factor towards the market consolidation, which took place during that time. There were only a few companies able to build such supercon magnets, most of them affiliated to or even owned by one of the major vendors. Magnets were expensive and hard to get which set a high bar to newcomers in the field while the original market leaders were bought up and eventually vanished. Diasonics sold their MR division to Toshiba, which eventually discontinued it in favor of their own product line. Technicare was sold to GE in 1985 and shut down a year later. Picker changed to Marconi, was later bought by Philips and quickly thereafter vanished.

### The intermediate years

In the early 80 s all scanners used simple spin echo sequences. The sequence ‘zoo’ in the early days was quite small. The preferred implementation was a dual echo sequence with one early echo for spin-density contrast and a late echo for T2-contrast. Gradient echo MRI was introduced in 1986 [38, 39] and immediately gained a lot of interest, and enabled novel applications like Cardiac MRI and MR-angiography [40–42].

RARE [43] was introduced at the same time as FLASH, but took a much longer time to find acceptance due to its sensitivity to eddy currents and became popular only in the early 90 s after the introduction of actively shielded gradients [44].

After the mid-90 s the MR-market appeared to be consolidated. With FLASH (GRASS, FFE,…) [38, 39] and RARE (TSE, FSE,…) [43] the main acquisition sequences had been established, shielded gradients took care of the eddy current issues of supercon magnets and phased array coils led to better image quality and improved versatility for signal reception. The basic and still used methods for contrast manipulation had been introduced. In addition to MR-angiography and flow imaging [45] this includes fat suppression/manipulation by chemical shift selective suppression (CHESS) [46], short TI inversion recovery (STIR) [47], and the Dixon technique [48], magnetization transfer [49], perfusion imaging with contrast agent [50] or by arterial spin labeling(ASL) [51] to name just a few. Diffusion MRI was already there [52] but had to await stronger gradients to become clinically relevant. Some of these methods showed inherent advantages at higher fields beyond the mere increase in SNR compared to lower fields. This includes especially MR-angiography (longer T1 leads to higher vessel signal and better background suppression), plus all techniques related to fat suppression/manipulation (higher chemical shift separation in Hz) as well as ASL (less T1-loss by longer T1), all of which added to the attraction of going to 1.5 T.

Already then the technology seemed to be mature and there was actually a perception that not much more new will come. In terms of field strength the development seemed to be aimed at higher and higher fields. The clinical low-field regime below 0.5 T was left to permanent magnet systems.

There was still an active scientific community working on low-cost MR.I at very low-fields. One way to make up for the low signal was field cycling, where spins are prepolarized at a higher field and measured at low-field.

This idea was already known in NMR spectroscopy and suggested by Al Macovski's group for use in MRI in 1993 [53]. The rationale for this was the insight that the polarizing magnet does not need to be very homogeneous, a field homogeneity in the order of a few percent is sufficient to generate homogeneous polarization. Thus the magnet can be rather cheap. Field cycling has been intensively used for measuring the field dependency of T1 and T2 for various tissues and contrast agents and there is an ongoing discussion that this field dependence may be the source of potentially relevant clinical contrast. A field-cycling scanner with a polarizing field of 0.4 T and a readout field of 0.027–0.2 T was used to generate ‘protein’ image contrast based on the cross-relaxation between protons and nitrogen at the quadrupole transition frequencies of nitrogen [54]. As an alternative approach a field modulation insert placed inside a clinical scanner was suggested [55]. In order to avoid strong coupling with the main coil, which may even lead the main magnet to quench, such inserts require extremely good B0-shielding and have typically a very small sample volume. Only quite recently a field cycling scanner for human applications has been realized, which works with field strength of up to 0.2 T [56]. Contrary to the original concept of using a high polarizing field, this particular system actually uses the highest field for data acquisition and the field is rapidly ramped down to measure the T1-dispersion at lower fields.

A recent review shows gives an excellent overview of the various technological approaches [57].

Another way to improve SNR at very low-field is to use superconducting quantum interference devices (SQUIDs) for signal detection, which was introduced to MRI in 1997 [58, 59] and often is used in conjunction with field cycling at very low-fields. Both principles are active fields of research to this day, but have—so far—failed to be translated into widespread clinical application.

For a long time the development of field strength in clinical MRI seemed to aim at higher and higher field strength, and for a long time low-field MRI was considered as a thing of the past—been there, done that. In 2003, 3 T became clinical and there was some expectation that history would repeat itself and 1.5 T would vanish, but this has not happened.

Permanent magnet systems with typical field strength of 0.2–0.4 T are still strong in the clinical market, although not very many publications or presentations at scientific meetings appear. Market leader Hitachi—who has recently merged with Fujifilm—has sold more than 7000 of its permanent magnet systems worldwide. High-end permanent magnet systems may include actively shielded gradients [60], and active shims and are capable to run advanced sequences like RARE (TSE, FSE,…), DTI/DWI, and EPI. Such additional components will, however, increase the cost of the system and, therefore, are used only sparsely especially given the fact that permanent magnet materials have become increasingly expensive while at the same time supercon magnets have become cheaper, so the cost advantage of permanent magnet systems has been shrinking.

### Low-field revival

A number of factors have come together to initiate a more widespread revival of low-field and ultra-low-field MRI. ‘Accessible MRI’ has been a buzzword emphasizing the fact that with all the fabulous progress which was been made, MRI is still only available to the privileged few living in the developed parts of developed countries and the huge majority of the population has no access to this modality, which is a cornerstone of current clinical care. There is hardly any patient entering a modern hospital today who will not get an MRI. It may discussed whether all these MRIs are really necessary, but that’s a different topic of discussion. Industry has become increasingly interested in branching off to new markets. In spite of the very long waiting times to get a MR-examination even where MRI is accessible, the market is perceived to be more or less saturated, and growth potential for ‘conventional’ 1.5 and 3 T scanners is seen mostly in countries far ahead in their development like China.

**Fig. 3:**
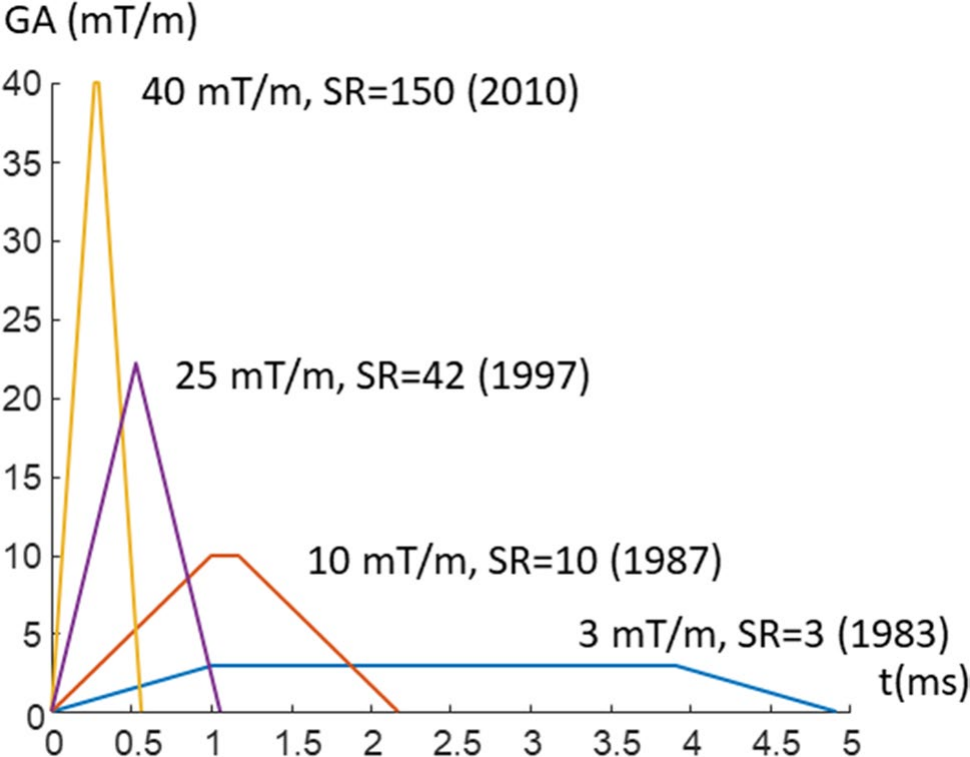
Gradient waveform for the maximum phase encoding step for gradient system of different maximum gradient amplitude and slew rate over the years

A lot of technological developments have taken place since low-field MRI has been left behind in the mid-90 s. The advent of parallel imaging has led to intense development in RF-coils, together with new developments in the receiver chain this has led to better signal-to-noise. Especially notable is the tremendous development in gradient hardware leading to much higher gradient amplitudes (GA) and slew rates (SR). This development had originally been driven in order to allow echo planar imaging applications like fMRI, but it also has a direct impact on the sampling efficiency of conventional scans. The first commercial MRI scanners had a maximum GA of ~ 3mT/m, which could be switched in ~ 1 ms corresponding to a slew rate of 3 T/m/s. In the mid-80 gradients with GA = 10–16 mT/m became available at still modest slew rates of 10 T/m/s. A new generation of gradient power amplifiers allowed an increase to GA = 25 mT/m and SR = 42 T/m/s in the mid-90 s [61]. Sampling efficiency in MRI is defined as the percentage of time spent on data acquisition compared to the total scan time. Conventional Fourier imaging requires some time to apply the RF-pulses and some time for the phase encoding gradient, during which no data acquisition takes place. Figure 3 illustrates the shape and duration of the maximum phase encoding gradient to encode for 1 mm spatial resolution for typical gradient systems used between 1983 and today. The time required for the phase encoding gradient directly impacts the sampling efficiency especially for commonly used fast sequences like gradient echoes (FLASH, GRASS, FFE,…) and RARE (TSE, FSE,…). The issue is acerbated by the fact that both types of sequences also require a phase encoding rewinder of equal duration. The diagram illustrates that for typical echo spacing of 10–20 ms in the early days a lot of time had to be spent for phase encoding and the sampling efficiency was low. The actual gradient amplitude for signal readout is not overly high, a scan with 1 mm resolution and an acquisition time of 5 ms requires only a gradient amplitude of 4.7 mT/m, but strong and especially fast gradient are essential to increase sampling efficiency.

**Fig. 4:**
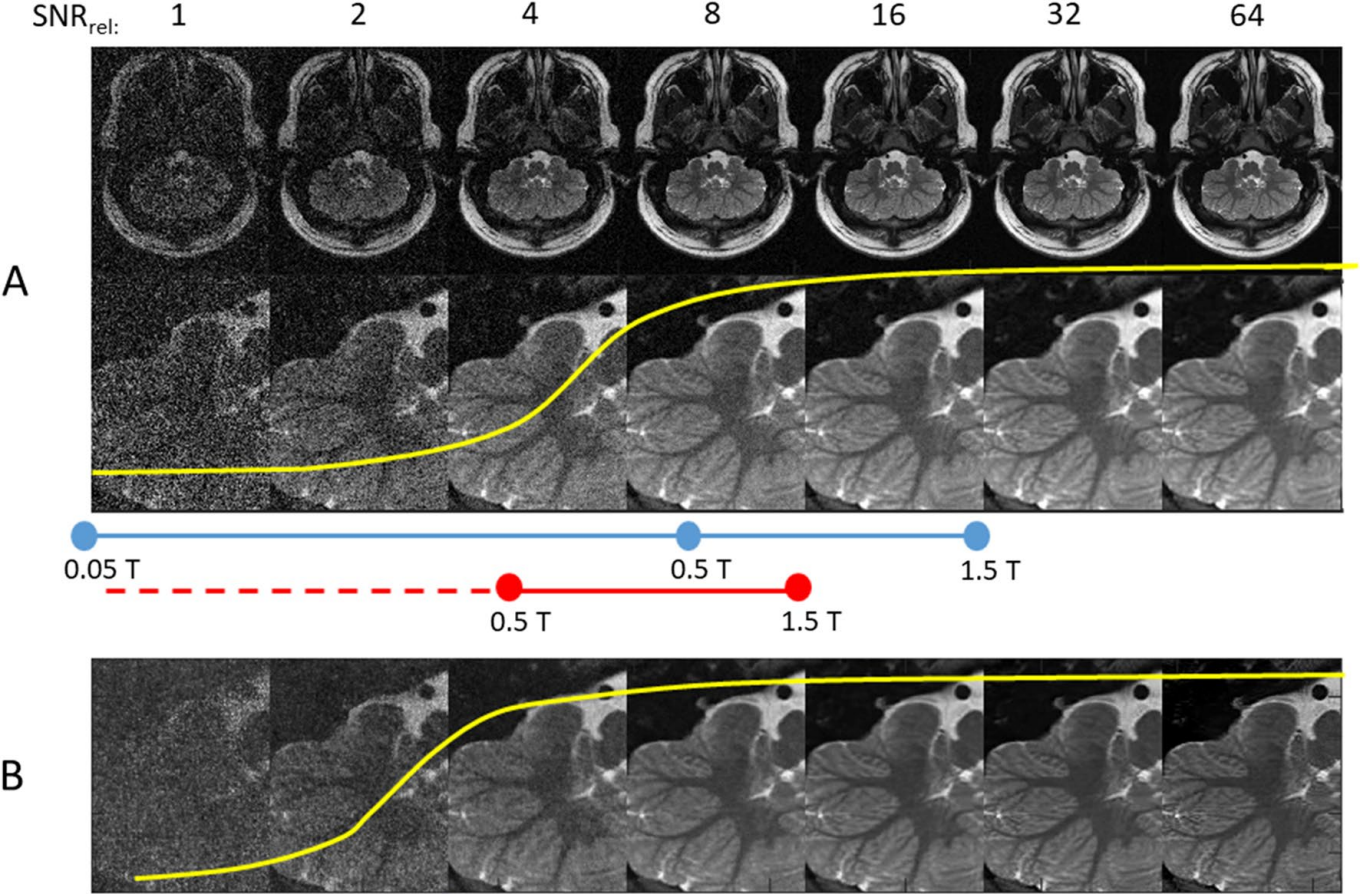
**A** Perceived image quality (indicated by the yellow line) vs. SNR shows a biphasic characteristic. In the noise dominated regime image quality is perceived as bad irrespective of the nominal SNR, in the signal dominated regime image quality does not get better with increased SNR. A very good image at 1.5 T will correspond to a still useful image at 0.5 T (blue line), if the image is already noisy at 1.5 T, image quality will become questionable at 0.5 T. **B** With a mild AI-based noise filter (Topaz Gigapixel^®^ filter, Topaz Labs, Dallas, USA) perceived image quality is already shifted to the left. Note that corresponding SNR at 0.05 T stays in the noise dominated regime

Since the increased signal-to-noise-ratio (SNR) at higher fields has been at the core of the discussion about high field MRI, it is worthwhile to take a look at image quality vs. SNR. Figure 4 shows that for a monotonous change in SNR the perceived image quality takes on a nearly biphasic character: In the noise dominated regime perceived image quality is unacceptable irrespective of the nominal SNR-value, in the signal dominated regime perceived image quality does not get better irrespective of SNR. This why SNR is an excellent measure for the performance of a given imaging sequence, but a poor measure of image quality. In the early 90 s images even at 1.5 T still looked somewhat noisy and spatial resolution was rarely better than 1 mm even in brain imaging. The only widely available means to improve SNR was low-pass filtering, which was not really acceptable. Therefore the only way to make up for the factor of 3 difference in SNR between 0.5 and 1.5 T was to increase the number of averages, which, however, quickly leads to unacceptable long acquisition times. All of this has changed dramatically: The underlying image quality has been considerably improved, in parts due to improvements in receiver hardware (coils, cable, preamps…), but also due to much improved image reconstruction and new and more flexible acquisition schemes.

Compressed sensing with iterative reconstruction allows to optimize SNR without increase in acquisition time and little or no loss in spatial resolution. Furthermore even in the pre-AI days images were routinely augmented using suitable post processing routines built into the reconstruction process and with AI noise has lost its terror (s. Figure 4B). Finally relaxation times are more favorable at lower fields—T1 gets shorter, T2 gets longer—both of which translate into better image quality in most commonly used sequences (especially RARE(TSE, FSE,…)) [43].

So from a methodological and technological standpoint the re-introduction of clinically viable low-field images with image quality in a similar ballpark as current 1.5 T-systems has really been there for many years. For some years sessions on low-cost, accessible MRI have been introduced at the Annual Meetings of ESMRMB and ISMRM and ISMRM workshops have been held in 2019 in Delhi and recently in 2022.

**Fig. 5:**
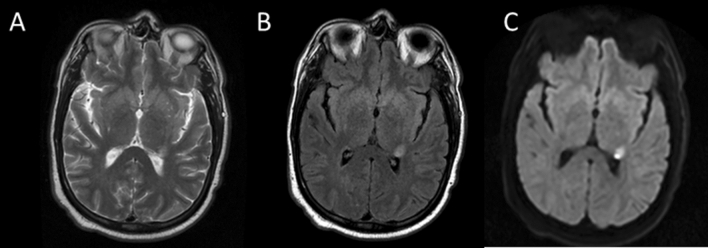
TSE (**A**), FLAIR (**B**) and DWI-EPI (**C**) images acquired on a dedicated point-of-care head scanner (Synaptive MRI, Synaptive Inc., Toronto, Canada) with 0.5 T field strength. Image parameters: TSE: 0.9 × 1 × 5 mm, 264 Hz/pix, TE = 86 ms, TR = 5550 ms, 4 avgs, 28 slices, 4:26 min; FLAIR: 1 × 0.9 × 5 mm, 192 Hz/pix, TE = 86 ms, TI = 1900 ms, TR = 5890 ms, 3 avgs, 28 slices, 4:26 min. DWI-EPI: 2 × 2 × 5 mm, 1500 Hz/pix, TR = 3940 ms, 6 avgs, 28 slices, 1:35 min (courtesy Jeff Stainsby, Syaptive Inc.)

**Fig. 6:**
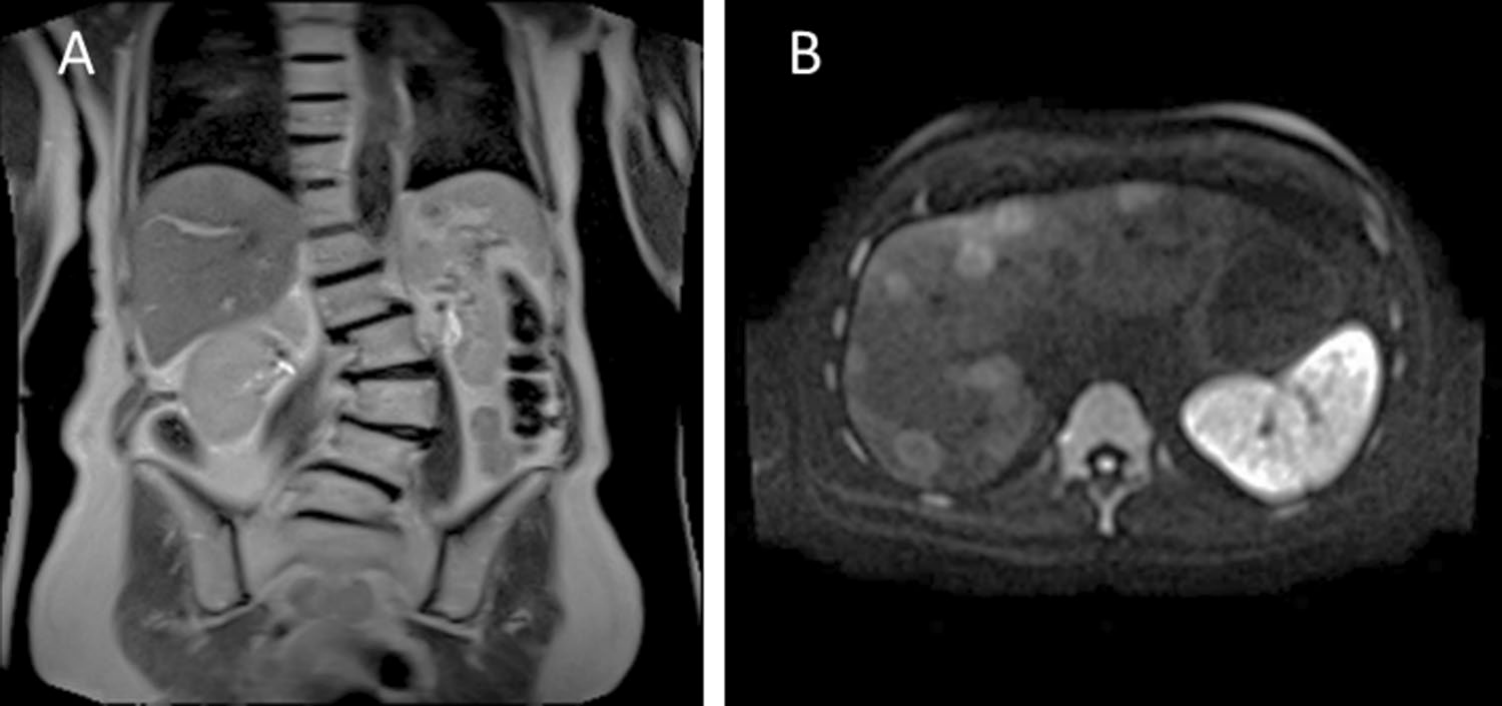
**A** Coronal T2 HASTE image of the abdomen and **B** DWI-EPI of the liver acquired at 0.55 T (FreeMax, Siemens; Erlangen, Germany). Imaging parameters: T2 HASTE: PAT 3, TR = 1000 ms, TE = 92 ms, 1.5 × 1.5 × 6.0mm^3^, TA: 3:07 min. DWI EPI: PAT 2, 1.6 × 1.6 × 6.0 mm^3^, 4:26 min, b-factor: 800 (courtesy University Hospital Erlangen)

In retrospect the time has been ripe for a new look at low-field MR. It still was a bold step, when in 2019 the team of Adrienne Campbell-Washburn ramped down their 1.5 T system to 0.55 T and demonstrated the very respectable image quality which can be achieved even with rather conservative means [62]. The importance of this bold step has been immediately recognized in an editorial published in the same issue [63] and the field has been thriving since then. Several other groups ramped down existing 1.5 T systems and the first commercial systems have been introduced by Synaptive Medical (0.5 T) and Siemens (0.55 T). Figures 5 and 6 illustrate the excellent image quality which be achieved. The enabling technology to allow ease-of-installation of such systems is the development of closed magnets, which do not need any Helium refill. Not having a quench pipe reduces installation cost and the lack of Helium refill makes operation much cheaper and feasible.

The development goals in this regime are clear: as follows to deliver diagnostic image quality with no compromise in examination time. The scanner makes up only a fraction of the operation cost of a MR-system, cost of personnel and infrastructure together with the need of high patient throughput very often outweigh the investment cost.

The primary goal for going to low-field was to achieve comparable image quality for similar protocols used at 1.5 T adapted to the different relaxation rates.

It was quickly realized that low-field MRI does not only achieve image quality at least comparable to 1.5 T, but also adds additional degrees of freedom to data acquisition. The considerably reduced susceptibility effect translate to very nice EPI-images with considerably reduced distortion even for abdominal imaging (see Figs. 4 and 5). It also allows to finally realize non-Cartesian sampling schemes like spirals, which have been around for nearly 50 years but never made it into clinical practice [64]. In the old days, when field strength was low, gradients were too slow to cover k-space in reasonable time, while with high fields (1.5 T and 3 T) susceptibility artifacts start to become a problem—although successful implementations have been demonstrated. At 0.55 T, susceptibility problems are much less an issue, but concomitant fields have to be dealt with, when gradients with high amplitude are being used [65, 66]. A further venue to explore are high-RF-sequences. Specific absorption rate at 0.55 T is very modest, so sequences requiring lots of RF—like magnetization transfer orT1-rho—offer much more flexibility. Naturally the full exploration of the opportunities at low-field requires appropriate hardware—sufficient gradient performance and RF power. Gradient slew rate is especially critical (s. Figure 3). Low slew rate will take away considerably more time and reduce the sampling efficiency. Since low-field systems are meant to be also low cost, an appropriate compromise needs to be found.

Ultralow-field MRI at field strength still an order of magnitude lower, has also found new interest. 0.05 T has actually been the field strength at which the early pioneers did their work, but the changes in pertinent technology since then are even more pronounced. Looking at Fig. 4 it is clear that there is no way to bring image quality into a comparable range as a 1.5 T or even 0.5 T system. So one has to take a much more radical approach, focusing on solving clinical problems rather than just trying to optimize image quality.

The first such systems were introduced ~ 2015. Matt Rosen used a rather conservative hardware design [67], but in 2018 published a radical AI-based approach to image reconstruction by introducing AutoMap, which avoids Fourier Transformation altogether by going from raw data directly to images [68]. Larry Wald and Jason Stockman used a more radical approach for their magnet [69]: Building on the insight that spatial encoding fields do not necessarily need to be linear they designed an ultra-compact Halbach magnet and used the field inhomogeneity for spatial encoding by rotating the magnet. Following this early work, other groups have followed with their own versions of ultralow-field systems [70, 71]. With the Hyperfine Swoop^®^ there is even a commercial system on the market and it will be interesting to see, where this development leads to. Such systems are not really comparable or even competitive to current clinical MRI systems, but are aimed at application scenarios not accessible to current MRI like remote areas [72, 73] or low-income countries. In terms of clinical usability and pricing they compete mainly with ultrasound, not clinical MRI. Therefore it does make sense to realize such systems to neuro applications, for everything else it will be hard to beat ultrasound. When it comes to bringing such systems to remote areas e.g. in rural Africa, where very often even ultrasound is not available, it will be a tough decision for potential customers whether they will really go for a MRI with a very narrow (but also very relevant) scope of applications or first invest in a much more versatile Ultrasound system at a similar price.

As part of the ISMRM Historical Archives Committee I organized a session on ‘MRI in India’ at the 2022 joint ISMRM-ESMRMB-meeting in London and of course the question came up whether such ultralow-field systems may be an option to deal with the immense clinical need there. I was quite astonished by the very vehement refusal of such an idea by the Indian experts at the meeting. They made it clear that yes, they urgently need more MRI, but they want MRI with current standard performance and not a cheap surrogate. This illustrates that this is a multifaceted issue and the use and perception of this intriguing technology may very much depend on many factors. Different settings will have different needs and the perception and acceptance will depend on more than just technological performance.

From looking at Fig. 4 it is clear that the gap in SNR between 0.05 T and 1.5 T is huge. Using measurement methods to optimize SNR (or better SNR per unit time) is of paramount importance. Therefore 3D-acquisition is the preferred acquisition scheme. It has been shown that the short T1 at low field allows for efficient 3D-acqusition using 3D-RARE techniques in reasonable acquisition times [71, 74]. With suitable gradient performance and RF-coils SMS-EPI may also be an option [70]. There has been a suggestion by M.Griswold that data acquisition with randomized flip angle and phase as used in MR fingerprinting shows a high mean average signal intensity [75]. Even with SNR-optimized acquisition schemes the image quality of primary images is still rather low at acquisition times of 5–10 min tolerable for clinical applications. Therefore the use of AI for noise filtering has become even more important than at higher fields. Recently Ed Wu has shown, that ULF-images can show image quality comparable to 3 T by using a noise filtering algorithm based on 3 T images from the Human Connectome Project database [76]. A common challenge with such extreme noise filtering approaches is the question, whether these approaches will be able to reliably detect pathologies, which were not part of the training datasets used to train the algorithms. In the old days radiologists were used to be able to ‘read-through’ noise, image artifacts, and other imperfections. At the same time they could make a good assessment of the reliability of their reading. With AI image reconstruction, images always look good and it is a much more tricky issue to assess the reliability of diagnosis.

These caveats notwithstanding ULF-MRI is a highly vibrant field-of-research, which has made tremendous progress within a few years. It is easy to get in since many groups have made the technical details of their developments public using an open source approach [77–81], so the field is open to new entrants without the necessity to re-develop all technical details.

## Conclusion

Without having any exact numbers I perceive that currently the majority of low-field and ultralow-field systems goes to institutions in the traditional developed and affluent regions. The aim of ‘democratizing’ MRI to bring into more remote areas is only slowly happening. It is nice to look into cost-efficient MR systems, but low cost for potential customers means low profit for vendors, so the business model is not so easy especially thinking about the cost for maintenance, training, infrastructure necessary for stable operation and—last but not least—the lack of qualified radiologists able to read the results. AI will definitely be an indispensable tool to make that happen.

For many years clinical MRI was running on only a few tracks: 1.5 and 3 T for day-to-day routine and 7 T for special applications like epilepsy. Now the ‘zoo’ of field strengths has considerably grown over the last few years. At the high-end spectrum 5 T has entered the scene and at the low and ultra-low-field range 0.5 T and 0.05 T have found interest. It is a mute issue to discuss about the optimum field strength, all field strength have their own flavor, challenges, and opportunities. From a developers point-of-view all field strengths are fun to work with and it is probably no coincidence, that many of the scientists working at low-field are also active at high and ultrahigh fields.

The Finnish have 40 words for snow. It will be a challenge for the future to find the right terminology for the wide range of current field strengths in clinical MRI.

## Data Availability

Not applicable.
